# Supplementary material for: Rapid Assessment of Therapeutic Empathy (RATE): Development of a universal single-item empathy scale and validation of the patient-reported version
Source: PLoS One. 2026 Sep 9;21(9):e0346994. doi: 10.1371/journal.pone.0346994 (PMC13557358; doi:10.1371/journal.pone.0346994)
Supplement: Supplementary file 1 [file pone.0346994.s001.pdf]

S1 Appendix. Questions used to screen digital exclusion and illiteracy based on previous research [1]

I can use online surveys on your mobile phone, computer, or another electronic device on my own (without asking for help from someone else).

Yes ☐

No ☐

References

1. Nelson LA, Pennings JS, Sommer EC, Popescu F, Barkin SL. A 3-item measure of digital health care literacy: development and validation study. JMIR Form Res. 2022;6(4):e36043. doi: 10.2196/36043.
