# Supplementary material for: Rapid Assessment of Therapeutic Empathy (RATE): Development of a universal single-item empathy scale and validation of the patient-reported version
Source: PLoS One. 2026 Sep 9;21(9):e0346994. doi: 10.1371/journal.pone.0346994 (PMC13557358; doi:10.1371/journal.pone.0346994)
Supplement: Supplementary file 2 [file pone.0346994.s002.pdf]

S2 Appendix. Table describing the components of therapeutic empathy

Supplementary table 1. Components of therapeutic empathy [1].

Component	Description
**Exploring**	Involves the practitioner actively seeking information about, and actively listening to, the patient's perspective. It includes having curiosity about the patient's wider background, and should be non-judgemental.
**Understanding**	After *exploring*, the practitioner develops their own *understanding* of the patient's perspective through cognitive processes. They use their imagination, logic, and reasoning to try to put themselves in the patient's shoes.
**Shared understanding**	To reach a *shared understanding* of the patient's perspective, the practitioner communicates their own *understanding* back to the patient to check its accuracy and correct it if necessary.
**Feeling**	Involves the practitioner *feeling* something in response to their *understanding* of the patient's circumstance (for example, of compassion or concern). This is not about the practitioner feeling the same emotions as the patient.
**Therapeutic action**	The caring behaviours performed by practitioners to help their patients. While this may involve establishing a care management plan or a course of treatment, it can also simply be actively listening to, and caring for the patient.
**Maintaining boundaries**	Throughout the empathic encounter, the practitioner must maintain a distinction between the "self" and the "other" such that their own emotions, experiences, and choices are recognised as being distinct from the patient's.

References

1. Howick J, Bennett-Weston A, Dudko M, Eva K. Uncovering the components of therapeutic empathy through thematic analysis of existing definitions. Patient Educ Couns. 2024:108596. doi: 10.1016/j.pec.2024.108596.
