# Supplementary material for: Rapid Assessment of Therapeutic Empathy (RATE): Development of a universal single-item empathy scale and validation of the patient-reported version
Source: PLoS One. 2026 Sep 9;21(9):e0346994. doi: 10.1371/journal.pone.0346994 (PMC13557358; doi:10.1371/journal.pone.0346994)
Supplement: Supplementary file 3 [file pone.0346994.s003.pdf]

S3 Appendix. Free-text questions used to elicit expert panel feedback for the assessment of content validity.

1. How easy to understand is the scale?
2. Do you have any suggestions for how we could make the scale clearer and easier to understand?
3. Are there terms or phrases that could be misunderstood or ambiguous?
4. How well do you think this version of the scale aligns with the following definition of therapeutic empathy? “Therapeutic empathy (empathy in the healthcare setting) involves listening, understanding someone’s situation and feelings, caring, and helping, all while respecting personal boundaries”
5. Do you think anything is missing from this version of the scale? (If so, please specify)
6. What, if anything, would you add or change to improve the scale and its ability to measure therapeutic empathy?
7. Please add any other comments you may have relating to this version of the scale.
