# Supplementary material for: Rapid Assessment of Therapeutic Empathy (RATE): Development of a universal single-item empathy scale and validation of the patient-reported version
Source: PLoS One. 2026 Sep 9;21(9):e0346994. doi: 10.1371/journal.pone.0346994 (PMC13557358; doi:10.1371/journal.pone.0346994)
Supplement: Supplementary file 4 [file pone.0346994.s004.pdf]

S4 Appendix: Topic guide for semi-structured cognitive interviews.

Please silently read each question. Once you have read a question, I would like you to mark your answer while verbalising the thought process involved with providing that answer.

Probes:

a. How did you come up with your answer?
b. Why have you given that answer?
c. Can you reword this question in your own words?
d. Is there a better way to ask this question?
e. What do you think this question is asking you?
f. What sort of things come to mind when you see the phrase: [insert phrase from questionnaire]?
g. What does [insert excerpt of question] mean to you?
h. Was that question easy or hard to answer?
i. I noticed that you hesitated, can you tell me why/what you were thinking?
