# Supplementary material for: Rapid Assessment of Therapeutic Empathy (RATE): Development of a universal single-item empathy scale and validation of the patient-reported version
Source: PLoS One. 2026 Sep 9;21(9):e0346994. doi: 10.1371/journal.pone.0346994 (PMC13557358; doi:10.1371/journal.pone.0346994)
Supplement: Supplementary file 5 [file pone.0346994.s005.pdf]

S5 Appendix: Question to assess the construct of clinical neutrality.

Adapted from Hojat et al. [1]

On a scale of 1 (not at all) to 9 (completely) please rate the extent to which the healthcare professional controlled expressions of emotional reactions towards you, whether their reactions are positive or negative.

References

1. Hojat M, Mangione S, Nasca TJ, Cohen MJ, Gonnella JS, Erdmann JB, et al. The Jefferson Scale of Physician Empathy: development and preliminary psychometric data. Educ Psychol Meas. 2001;61(2):349-65. doi: 10.1177/00131640121971158.
