# Supplementary material for: Rapid Assessment of Therapeutic Empathy (RATE): Development of a universal single-item empathy scale and validation of the patient-reported version
Source: PLoS One. 2026 Sep 9;21(9):e0346994. doi: 10.1371/journal.pone.0346994 (PMC13557358; doi:10.1371/journal.pone.0346994)
Supplement: Supplementary file 6 [file pone.0346994.s006.pdf]

# S6 Appendix. Initial versions of the universal single-item RATE scale to measure therapeutic empathy

## Patient-reported version

### Version 1. Pictorial

People who help you with your health, like doctors and nurses, show they care for you by:

- Listening
- Asking what matters to you
- Being kind
- Helping you

When they do these things, we say they are being "empathic". Think about the last time you saw someone to help you with your health. Point to the smiley face that shows how empathic they were:

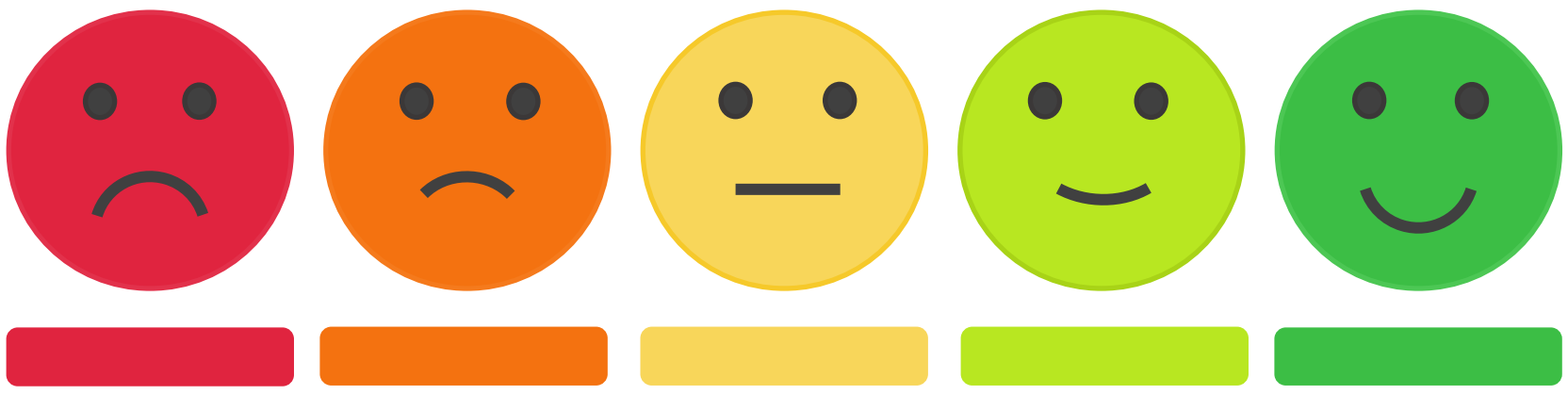

### Version 2. Text-based

Healthcare workers, like doctors and nurses, show empathy for you by:

- Listening carefully to you
- Noticing and understanding your feelings
- Asking what matters to you
- Being kind
- Helping you
- Making you feel comfortable

Think about the last time you saw a healthcare worker. How empathic were they?

| | | | | |
|---|---|---|---|---|
| 1. Not at all empathic | 2. A little empathic | 3. Somewhat empathic | 4. Very empathic | 5. Extremely empathic |

## Practitioner-reported version

### Version 1. Pictorial

Healthcare practitioners show empathy for their patients by:

- Listening
- Asking what matters to them
- Being kind
- Helping them

Think about the last time you saw a patient. Point to the smiley face that shows how empathic you were:

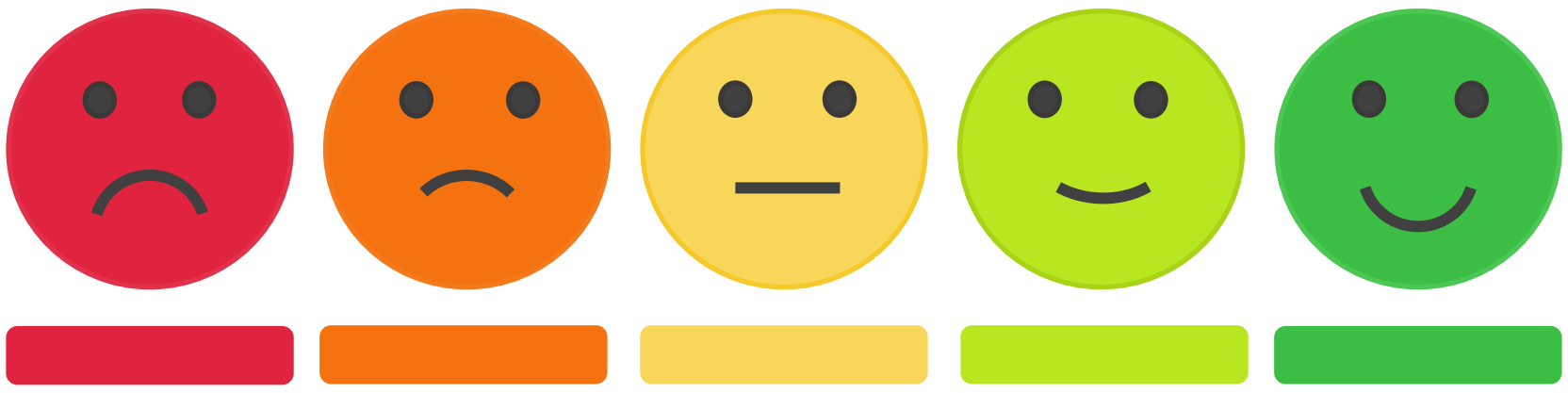

### Version 2. Text-based

Healthcare practitioners show empathy for their patients by:

- Listening carefully to them
- Noticing and understanding their feelings
- Asking what matters to them
- Being kind
- Helping them
- Making them feel comfortable

Think about the last time you saw a patient. How empathic were you?

| | | | | |
|---|---|---|---|---|
| 1. Not at all empathic | 2. A little empathic | 3. Somewhat empathic | 4. Very empathic | 5. Extremely empathic |

## Observer-reported versions

### Version 1. Pictorial

Healthcare workers, like doctors and nurses, show empathy for their patients by:

- Listening
- Asking what matters to them
- Being kind
- Helping them

Think about the last time you saw a healthcare worker interact with a patient. Point to the smiley face that shows how empathic they were:

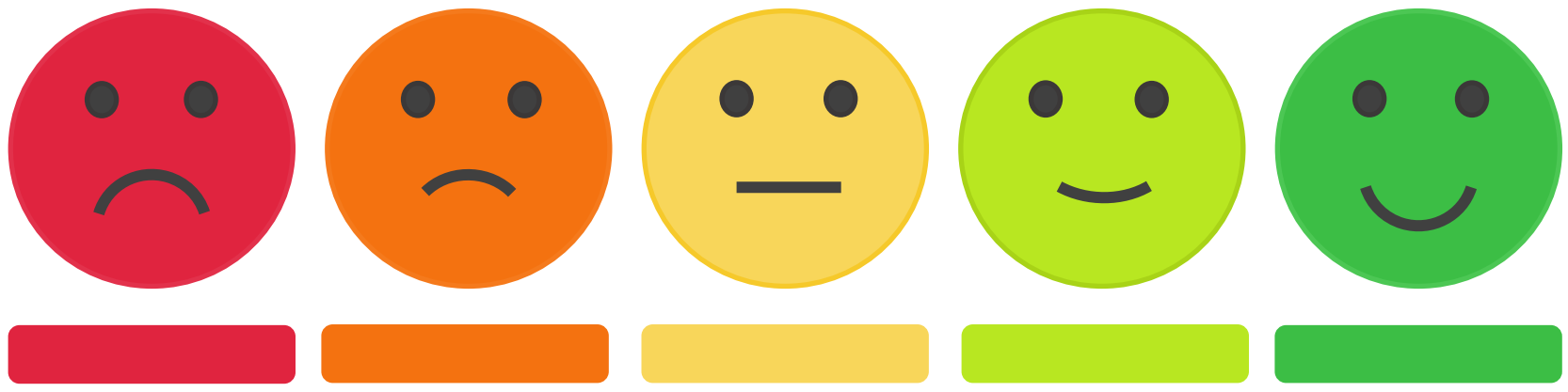

### Version 2. Text-based

Healthcare workers, like doctors and nurses, show empathy for their patients by:

- Listening carefully to them
- Noticing and understanding their feelings
- Asking what matters to them
- Being kind
- Helping them
- Making them feel comfortable

Think about the last time you saw a healthcare worker interact with a patient. How empathic were they?

| | | | | |
|---|---|---|---|---|
| 1. Not at all empathic | 2. A little empathic | 3. Somewhat empathic | 4. Very empathic | 5. Extremely empathic |

## Student-reported versions

### Version 1. Pictorial

Healthcare practitioners show empathy for their patients by:

- Listening
- Asking what matters to them
- Being kind
- Helping them

Think about the last time you saw a patient. Point to the smiley face that shows how empathic you were:

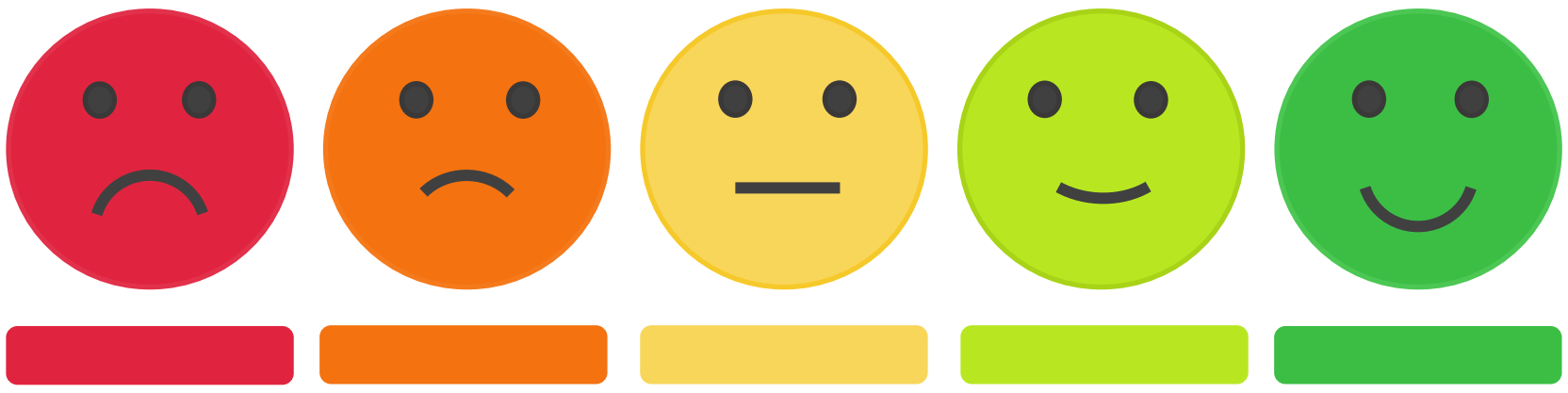

### Version 2. Text-based

Healthcare practitioners show empathy for their patients by:

- Listening carefully to them
- Noticing and understanding their feelings
- Asking what matters to them
- Being kind
- Helping them
- Making them feel comfortable

Think about the last time you saw a patient. How empathic were you?

| | | | | |
|---|---|---|---|---|
| 1. Not at all empathic | 2. A little empathic | 3. Somewhat empathic | 4. Very empathic | 5. Extremely empathic |
