# Supplementary material for: Rapid Assessment of Therapeutic Empathy (RATE): Development of a universal single-item empathy scale and validation of the patient-reported version
Source: PLoS One. 2026 Sep 9;21(9):e0346994. doi: 10.1371/journal.pone.0346994 (PMC13557358; doi:10.1371/journal.pone.0346994)
Supplement: Supplementary file 7 [file pone.0346994.s007.pdf]

S7 Appendix. Results of readability tests for initial versions of the RATE scale

Supplementary table 2. Readability scores for the initial versions of the universal single-item therapeutic empathy scale.

Scale version	Flesh-Kincaid Grade Level	Flesch Reading Ease
Patient-reported (smiley-face version)	2.8	88.8
Patient-reported (text-based version)	6.5	57.7
Practitioner-reported (smiley-face version)	5.4	66.0
Practitioner-reported (text-based version)	7.5	49.0
Observer-reported (smiley-face version)	4.7	73.1
Observer-reported (text-based version)	6.7	57.1
Student-reported (smiley-face version)	5.4	66.0
Student-reported (text-based version)	7.5	49.0
