# Supplementary material for: Rapid Assessment of Therapeutic Empathy (RATE): Development of a universal single-item empathy scale and validation of the patient-reported version
Source: PLoS One. 2026 Sep 9;21(9):e0346994. doi: 10.1371/journal.pone.0346994 (PMC13557358; doi:10.1371/journal.pone.0346994)
Supplement: Supplementary file 8 [file pone.0346994.s008.pdf]

S8 Appendix. Participant characteristics: Assessment of content validity

Supplementary table 3. Characteristics of expert panel members participating in assessment of content validity.

Participant ID code	Age backet (years)	Ethnicity	Role
**01**	≥70	White	Patient representative and advocate
**02**	50-59	White	Healthcare professional, researcher, and educator
**03**	40-49	Asian or Asian British	Healthcare professional, researcher, and educator
**04**	50-59	White	Patient representative and advocate
**05**	60-69	White	Healthcare professional, researcher, and educator
**06**	50-59	White	Healthcare educator and researcher
**07**	50-59	Other ethnic group	Healthcare educator and researcher
**08**	60-69	White	Healthcare professional, researcher, and educator
**09**	50-59	White	Healthcare professional and educator
