# Supplementary material for: Rapid Assessment of Therapeutic Empathy (RATE): Development of a universal single-item empathy scale and validation of the patient-reported version
Source: PLoS One. 2026 Sep 9;21(9):e0346994. doi: 10.1371/journal.pone.0346994 (PMC13557358; doi:10.1371/journal.pone.0346994)
Supplement: Supplementary file 9 [file pone.0346994.s009.pdf]

S9 Appendix. Participant feedback and modifications made to the RATE scales at each stage of development

Supplementary table 4. Modifications made based on feedback from expert panel and cognitive interviews.

Feedback	Modification to scale
**Expert panel feedback during assessment of content validity**	
The description of therapeutic empathy could be refined to better align with the definition [11] underpinning the scale, by including a statement to capture the component "maintaining boundaries".	Added a statement about "respecting patients' choices" to the description of therapeutic empathy to capture the component of "maintaining boundaries".
The phrase "helping you/their patients" could be expanded to more clearly describe the ways in which practitioners help patients when practicing therapeutic empathy.	Expanded the statement "helping you/their patients" to "helping you/them understand their health".
The statement "asking what matters to you/them" could be re-phrased to better capture the need for understanding as a central component of therapeutic empathy, and to avoid possible confusion with the sentence "asking what is the matter *with* you/them".	Changed "asking what matters you you/them" to "understanding what is important to you/them".
References to particular types of healthcare practitioner (for example, doctor and nurse) may bias responses by encouraging participants to complete the scale about these practitioners alone, at the expense of others.	Removed any reference to specific types of healthcare practitioner.
**Stakeholder feedback in round 1 of cognitive interviews**	
The statement "helping you/them understand their health" is too narrow, and precludes consideration of other forms of help provided by practitioners during an empathic interaction (for example, prescribing a treatment, validating someone's feelings, or getting them a cup of tea).	Changed "helping you/them understand your health" back to "helping you/them" to account for the breadth of helping behaviours provided by practitioners when practicing therapeutic empathy.
The active nature of "listening to you/them" should be emphasised as a central aspect of therapeutic empathy.	Added the word "carefully" to the statement "listening to you/them" so that it becomes "listening carefully to you/them".

Feedback	Modification to scale
The wording of the text-based scale could be softened and simplified for two reasons; first, few participants thought they would rate anyone (including themselves) as being “extremely empathic”, limiting response options. Second, the word “empathic” was not Plain English, reducing the scales accessibility.	Changed the text-based scale from “not at all empathic” to “extremely empathic”, to “poor” to “excellent”. This required a change in phrasing to “how good were they at showing empathy?” instead of “how empathic were they?”.
**Stakeholder feedback in round 2 of cognitive interviews**	
Use of the words “show” or “showing” in relation to the demonstration of therapeutic empathy presents it as a superficial construct. In participants’ experience, therapeutic empathy required practitioners to both *be* and *behave* empathically.	Adopted the following phrasing to avoid using the word “show” or “showing” in relation to the practice of therapeutic empathy: “In healthcare, empathy means that healthcare workers…” and “how good were they at doing these things?”.
The statement “respect your/their choices” could be expanded to “respect your/their views and choices” to better capture the meaning of therapeutic empathy and account for scenarios in which patients might have limited choice.	Expanded the phrase “respect your/their choices” could be expanded to “respect your/their views and choices”.
The scale could be revised to more clearly define empathy; while the behaviours involved in therapeutic empathy had been listed, several participants thought that this was not clearly a definition of the construct.	Rephrased the opening sentence of the scale to “empathy means that…” to make it clear that the list of behaviours that follows is how empathy is defined.
Adding a free-text comments box would enable participants to add more information about the interaction they are basing their rating on. This was considered particularly important given the single-item nature of the scale.	Added a free-text, optional comments box.
**Stakeholder feedback in round 3 of cognitive interviews**	
Adding numbers to the pictorial, smiley-face version of the scale might enhance its accessibility in paper- and web-based formats, supporting selection of the relevant response option.	Added numbers to the pictorial version of the scale.
The phrase “understand what is important to you/them” could be rephrased to better capture the fact that therapeutic empathy is about understanding the whole person, including their wider circumstances and feelings.	Rephrased the statement “understand what is important to you/them” to “understand your/their situation and feelings”.
