# Supplementary material for: Rapid Assessment of Therapeutic Empathy (RATE): Development of a universal single-item empathy scale and validation of the patient-reported version
Source: PLoS One. 2026 Sep 9;21(9):e0346994. doi: 10.1371/journal.pone.0346994 (PMC13557358; doi:10.1371/journal.pone.0346994)
Supplement: Supplementary file 10 [file pone.0346994.s010.pdf]

# S10 Appendix. Participant characteristics: Pre-testing the RATE scale in cognitive interviews

Eighteen patients and carers represented lived experiences of long-term mental illness, acute urinary infections, neurodiversity, long-term conditions, substance abuse and homelessness, and lifelong disability. Nine healthcare practitioners and educators represented various healthcare professions, including medicine, nursing, midwifery, physiotherapy, pharmacy, and operating department practice. Eight healthcare students represented medicine, nursing, midwifery, and physiotherapy courses. Participants' characteristics are summarised in Supplementary table 5.

**Supplementary table 5. Characteristics of cognitive interview participants to pre-test the scale.**

| Participant ID code | Age bracket (years) | Ethnicity | Role | Version(s) of the scale pre-tested |
|---|---|---|---|---|
| **Round 1 of Cognitive Interviews** | | | | |
| **10** | 30-39 | White | Patient | Patient-reported |
| **11** | 50-59 | Asian or Asian British | Patient/Carer | Patient-reported; Observer-reported |
| **12** | 60-69 | White | Patient | Patient-reported |
| **13** | 50-59 | White | Patient | Patient-reported |
| **14** | 18-29 | Asian or Asian British | Patient | Patient-reported |
| **15** | 60-69 | Asian or Asian British | Patient/Carer | Patient-reported; Observer-reported |
| **16** | 50-59 | White | Healthcare practitioner/Educator | Practitioner-reported; Observer-reported |
| **17** | 50-59 | White | Healthcare practitioner/Educator | Practitioner-reported; Observer-reported |
| **18** | 50-59 | White | Healthcare practitioner/Educator | Practitioner-reported; Observer-reported |
| **19** | 30-39 | White | Healthcare practitioner/Educator | Practitioner-reported; Observer-reported |

| Participant ID code | Age bracket (years) | Ethnicity | Role | Version(s) of the scale pre-tested |
|---|---|---|---|---|
| **20** | 30-39 | White | Healthcare practitioner/Educator | Practitioner-reported; Observer-reported |
| **21** | 40-49 | Asian or Asian British | Healthcare practitioner/Educator | Practitioner-reported; Observer-reported |
| **22** | 18-29 | White | Healthcare student | Student-reported |
| **23** | 18-29 | Asian or Asian British | Healthcare student | Student-reported |
| **24** | 18-29 | Asian or Asian British | Healthcare student | Student-reported |
| **Round 2 of Cognitive Interviews** | | | | |
| **25** | 18-29 | Black, Black British, Caribbean or African | Patient/Carer | Patient-reported; Observer-reported |
| **26** | 50-59 | White | Patient | Patient-reported |
| **27** | 70-79 | White | Patient | Patient-reported |
| **28** | 50-59 | White | Patient | Patient-reported |
| **29** | 80-89 | White | Carer | Observer-reported |
| **30** | 50-59 | White | Carer | |
| **31** | 40-49 | White | Healthcare practitioner/Educator | Practitioner-reported; Observer-reported |
| **32** | 18-29 | Black, Black British, Caribbean or African | Healthcare student | Student-reported |
| **33** | 18-29 | White | Healthcare student | Student-reported |
| **34** | 18-29 | Black, Black British, Caribbean or African | Healthcare student | Student-reported |
| **Round 3 of Cognitive Interviews** | | | | |
| **35** | 60-69 | White | Patient/Carer | Patient-reported; Observer-reported |
| **36** | 40-49 | White | Patient | Patient-reported |
| **37** | 50-59 | White | Carer | Observer-reported |
| **38** | 40-49 | White | Patient | Patient-reported |
| **39** | 50-59 | White | Patient | Patient-reported |
| **40** | 40-49 | White | Patient | Patient-reported |

| Participant ID code | Age bracket (years) | Ethnicity | Role | Version(s) of the scale pre-tested |
|---|---|---|---|---|
| **41** | 50-59 | White | Healthcare practitioner/Educator | Practitioner-reported; Observer-reported |
| **42** | 40-49 | Mixed or multiple ethnic groups | Healthcare practitioner/Educator | Practitioner-reported; Observer-reported |
| **43** | 18-29 | White | Healthcare student | Student-reported |
| **44** | 18-29 | Asian or Asian British | Healthcare student | Student-reported |
