# Supplementary material for: Rapid Assessment of Therapeutic Empathy (RATE): Development of a universal single-item empathy scale and validation of the patient-reported version
Source: PLoS One. 2026 Sep 9;21(9):e0346994. doi: 10.1371/journal.pone.0346994 (PMC13557358; doi:10.1371/journal.pone.0346994)
Supplement: Supplementary file 11 [file pone.0346994.s011.pdf]

S11 Appendix. Final versions of the universal single-item RATE scales

Patient-reported versions

Version 1. Pictorial

In healthcare, empathy means that healthcare workers:

- Listen carefully to you
- Understand your situation and feelings
- Are kind and caring
- Help you
- Respect your views and choices

Think about the last time you saw a healthcare worker. Choose the smiley face that shows how good they were at doing these things:

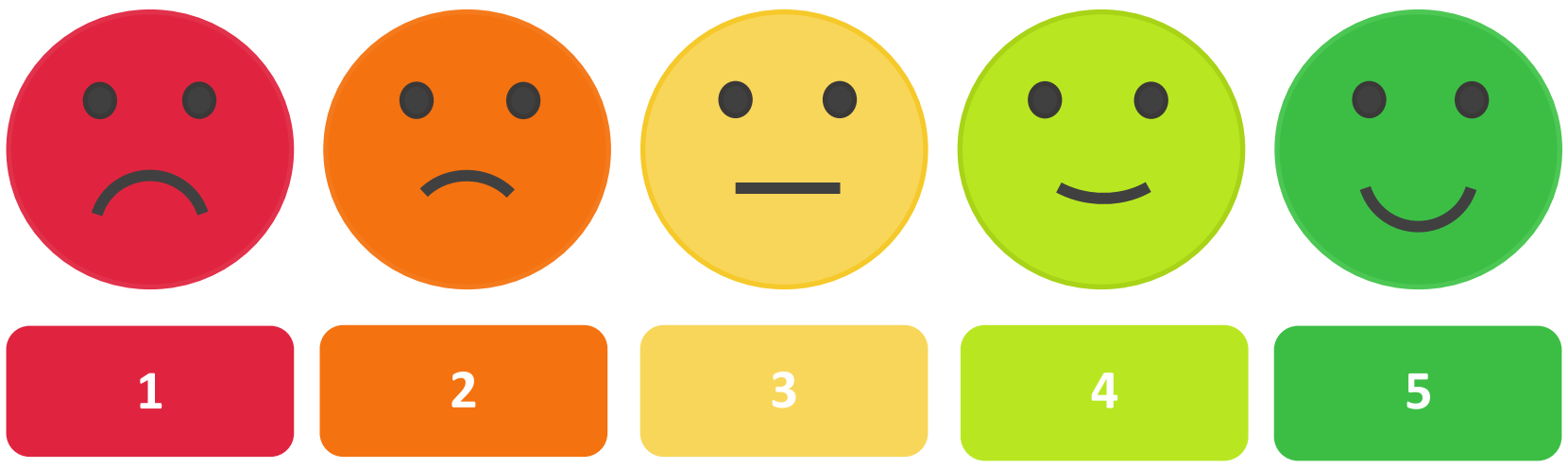

If you have any comments about the last time you saw a healthcare worker, please write them in the box below:

Version 2. Text-based

In healthcare, empathy means that healthcare workers:

- Listen carefully to you
- Understand your situation and feelings
- Are kind and caring
- Help you
- Respect your views and choices

Think about the last time you saw a healthcare worker. How good were they at doing these things?

1. Poor	2. Fair	3. Good	4. Very Good	5. Excellent

If you have any comments about the last time you saw a healthcare worker, please write them in the box below:

Practitioner-reported versions

Version 1. Pictorial

In healthcare, empathy means that healthcare practitioners:

- Listen carefully to their patients
- Understand their patients’ situation and feelings
- Are kind and caring
- Help their patients
- Respect their patients’ views and choices

Think about the last interaction you had with a patient. Select the smiley face that shows how good you were at doing these things:

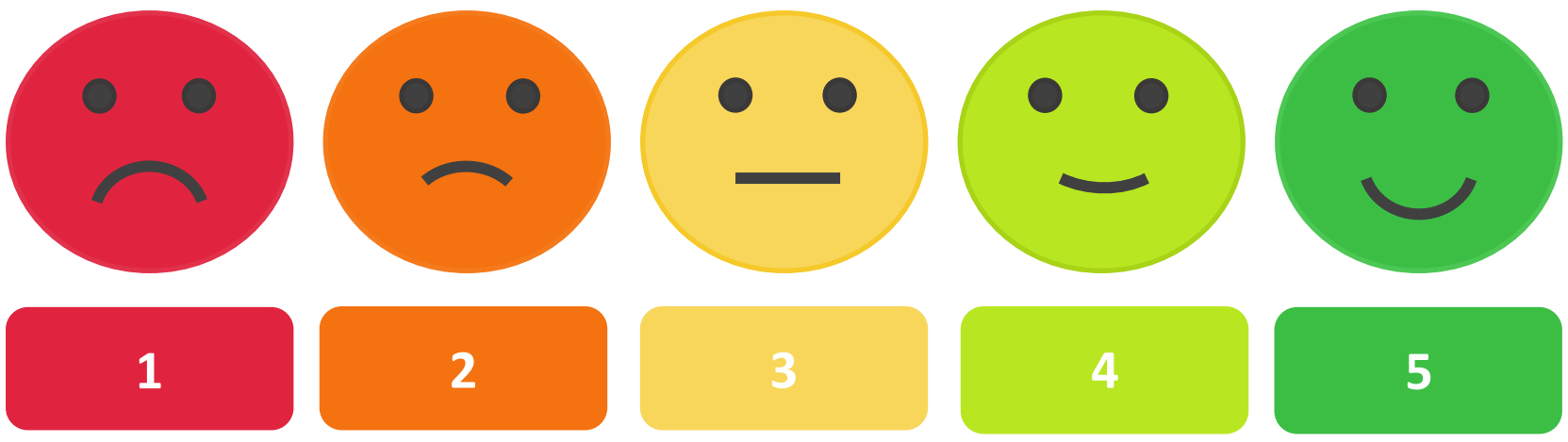

If you have any comments about the last interaction you had with a patient, please write them in the box below:

Version 2. Text-based

In healthcare, empathy means that healthcare practitioners:

- Listen carefully to their patients
- Understand their patients' situation and feelings
- Are kind and caring
- Help their patients
- Respect their patients' views and choices

Think about the last interaction you had with a patient. How good were you at doing these things?

1. Poor	2. Fair	3. Good	4. Very Good	5. Excellent

If you have any comments about the last interaction you had with a patient, please write them in the box below:

Observer-reported versions

Version 1. Pictorial

In healthcare, empathy means that healthcare practitioners:

- Listen carefully to their patients
- Understand their patients' situation and feelings
- Are kind and caring
- Help their patients
- Respect their patients' views and choices

Think about the patient-practitioner interaction you observed. Select the smiley face that shows how good the (student) practitioner was at doing these things:

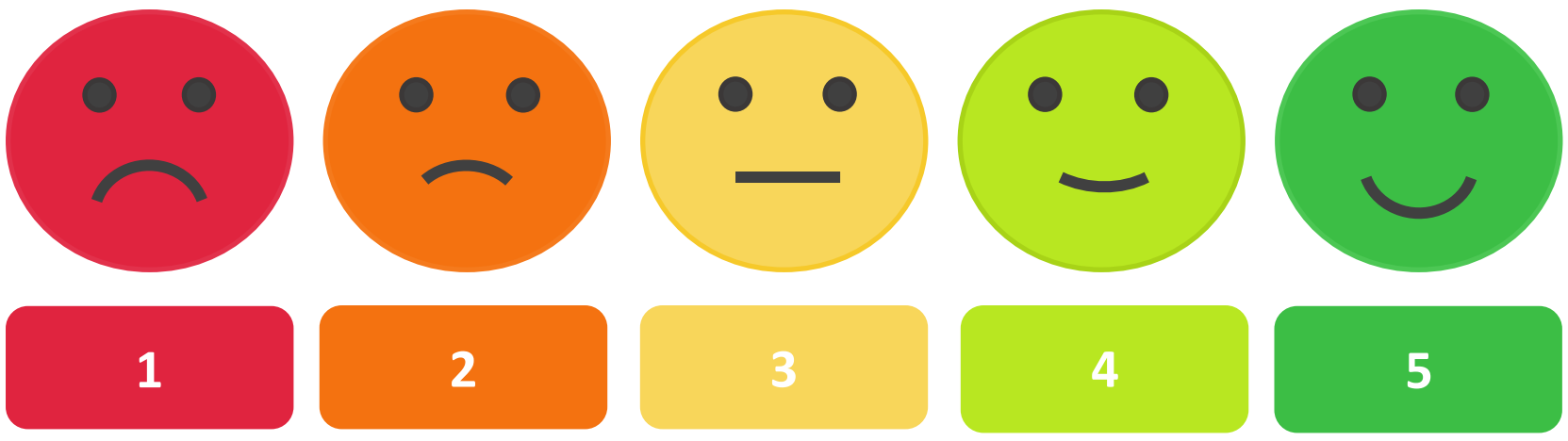

If you have any comments about the interaction you just observed, please write them in the box below:

Version 2. Text-based

In healthcare, empathy means that healthcare practitioners:

- Listen carefully to their patients
- Understand their patients' situation and feelings
- Are kind and caring
- Help their patients
- Respect their patients' views and choices

Think about the patient-practitioner interaction you observed. How good was the (student) practitioner at doing these things?

1. Poor	2. Fair	3. Good	4. Very Good	5. Excellent

If you have any comments about the interaction you just observed, please write them in the box below:

Student-reported versions

Version 1. Pictorial

In healthcare, empathy means that healthcare practitioners:

- Listen carefully to their patients
- Understand their patients' situation and feelings
- Are kind and caring
- Help their patients
- Respect their patients' views and choices

Think about the last interaction you had with a patient. Select the smiley face that shows how good you were at doing these things:

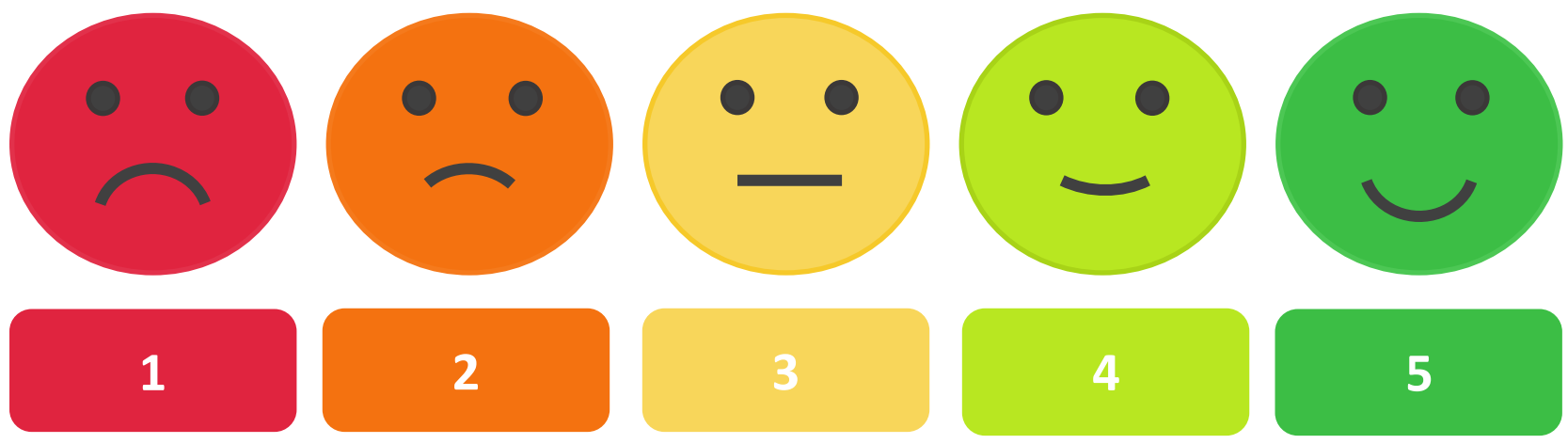

If you have any comments about the last interaction you had with a patient, please write them in the box below:

Version 2. Text-based

In healthcare, empathy means that healthcare practitioners:

- Listen carefully to their patients
- Understand their patients' situation and feelings
- Ask what is important to their patients
- Help their patients
- Respect their patients' views and choices

Think about the last interaction you had with a patient. How good were you at doing these things?

1. Poor	2. Fair	3. Good	4. Very Good	5. Excellent

If you have any comments about the last interaction you had with a patient, please write them in the box below:

