# Supplementary material for: Rapid Assessment of Therapeutic Empathy (RATE): Development of a universal single-item empathy scale and validation of the patient-reported version
Source: PLoS One. 2026 Sep 9;21(9):e0346994. doi: 10.1371/journal.pone.0346994 (PMC13557358; doi:10.1371/journal.pone.0346994)
Supplement: Supplementary file 12 [file pone.0346994.s013.docx]

Rapid Assessment of Therapeutic Empathy (RATE): Development of a universal single-item empathy scale and validation of the patient-reported version

# Response to Reviewers

| **Editor/reviewer comment** | **Author response and location of revision in manuscript** |
| --- | --- |
| **Journal requirements:**  1. When submitting your revision, we need you to address these additional requirements.  Please ensure that your manuscript meets PLOS ONE's style requirements, including those for file naming. | Thank you. We have made changes to ensure that our title page and manuscript are formatted to PLOS ONE’s style requirements. |
| **Journal requirements:**  2. We note that the grant information you provided in the ‘Funding Information’ and ‘Financial Disclosure’ sections do not match.    When you resubmit, please ensure that you provide the correct grant numbers for the awards you received for your study in the ‘Funding Information’ section. | Thank you. We have updated the Financial Disclosure section of our submission form as per the guidance in the point below, so it now reads as follows: “Jeremy Howick, Amber Bennett-Weston, Benita Iyadi-Wilson, and Patience Otoo were funded by the Stoneygate Trust (no grant reference available). Kamlesh Khunti is funded by the National Institute for Health Research (no grant reference available). Daya Narwal was funded by the National Institute for Health Research (grant reference: NIHR305951). John Maltby and Char Leung completed this work during the usual course of their employment at the University of Leicester and did not receive additional funding for this study. The funders had no role in developing the protocol. There was no additional external funding received for this study.”  We have checked the Funding Information section of our submission form and the grant numbers listed there (or absence of grant numbers as most external funding did not receive a grant number) match with what is listed in our Financial Disclosure statement. |
| **Journal requirements:**  3. Thank you for stating in your Funding Statement:  JH, ABW, BIW, and PO are funded by the Stoneygate Trust (no grant reference). KK is supported by the National Institute for Health Research (NIHR; no grant reference). DN was funded by NIHR (grant reference: NIHR305951). The funders had no role in developing the protocol.    Please provide an amended statement that declares *all* the funding or sources of support (whether external or internal to your organization) received during this study, as detailed online in our guide for authors at <http://journals.plos.org/plosone/s/submit-now>.  Please also include the statement “There was no additional external funding received for this study.” in your updated Funding Statement.  Please include your amended Funding Statement within your cover letter. We will change the online submission form on your behalf. | Thank you. We have updated our funding statement as follows, and included this in our cover letter:  Jeremy Howick, Amber Bennett-Weston, Benita Iyadi-Wilson, and Patience Otoo were funded by the Stoneygate Trust (no grant reference available). Kamlesh Khunti is funded by the National Institute for Health Research (no grant reference available). Daya Narwal was funded by the National Institute for Health Research (grant reference: NIHR305951). John Maltby and Char Leung completed this work during the usual course of their employment at the University of Leicester and did not receive additional funding for this study. The funders had no role in developing the protocol. There was no additional external funding received for this study. |
| **Journal requirements:**  4. Please expand the acronym “JH, ABW, BIW, PO KK and DN” (as indicated in your financial disclosure) so that it states the name of your funders in full.  This information should be included in your cover letter; we will change the online submission form on your behalf. | Thank you. As per our response above, to points 2 and 3, we have revised our Financial Disclosure and Funding statements to expand all acronyms, and ensure clarity. The acronyms noted here are in fact author’s initials, which we have now expanded:  “Jeremy Howick, Amber Bennett-Weston, Benita Iyadi-Wilson, and Patience Otoo were funded by the Stoneygate Trust (no grant reference available). Kamlesh Khunti is funded by the National Institute for Health Research (no grant reference available). Daya Narwal was funded by the National Institute for Health Research (grant reference: NIHR305951). John Maltby and Char Leung completed this work during the usual course of their employment at the University of Leicester and did not receive additional funding for this study. The funders had no role in developing the protocol. There was no additional external funding received for this study.” |
| **Journal requirements:**  5. We note that you have indicated that there are restrictions to data sharing for this study. For studies involving human research participant data or other sensitive data, we encourage authors to share de-identified or anonymized data. However, when data cannot be publicly shared for ethical reasons, we allow authors to make their data sets available upon request. For information on unacceptable data access restrictions, please see <http://journals.plos.org/plosone/s/data-availability#loc-unacceptable-data-access-restrictions>.  Before we proceed with your manuscript, please address the following prompts:  a) If there are ethical or legal restrictions on sharing a de-identified data set, please explain them in detail (e.g., data contain potentially identifying or sensitive patient information, data are owned by a third-party organization, etc.) and who has imposed them (e.g., a Research Ethics Committee or Institutional Review Board, etc.). Please also provide contact information for a data access committee, ethics committee, or other institutional body to which data requests may be sent.  b) If there are no restrictions, please upload the minimal anonymized data set necessary to replicate your study findings to a stable, public repository and provide us with the relevant URLs, DOIs, or accession numbers. Please see <http://www.bmj.com/content/340/bmj.c181.long> for guidelines on how to de-identify and prepare clinical data for publication. For a list of recommended repositories, please see <https://journals.plos.org/plosone/s/recommended-repositories>. You also have the option of uploading the data as Supporting Information files, but we would recommend depositing data directly to a data repository if possible.  Please update your Data Availability statement in the submission form accordingly. | Thank you. Please see our responses below.   a) Our dataset comprises a combination of quantitative and qualitative data from three participant groups: expert panellists (n=9), key stakeholders from each of our respondent groups including patients, practitioners, healthcare educators, carers, and healthcare students (n=35), and patients (n=521). Data from expert panellists includes age, gender, ethnicity, professional role and area of expertise, geographic location, and both Likert-scale scores and free-text comments to assess the relevance of our newly developed Rapid Assessment of Therapeutic Empathy (RATE) scale. Data from key stakeholders from each respondent group includes age, gender, ethnicity, professional role and area of expertise, geographic location, and where relevant health condition, along with interview transcripts describing their thought processes as they complete the newly developed RATE scale, which involves recalling recent healthcare appointments that are likely to be identifying. Data from patients (n=521) includes age, gender, ethnicity, geographic location, details about their last healthcare consultation including the practitioner they saw and where it took place, details about their health condition and/or disability, scores on the RATE scales, the Consultation and Relational Empathy (CARE) measure, and a measure of clinical neutrality. The data are not owned by a third-party organisation.   There are ethical restrictions on sharing our de-identified data set, imposed by the NHS Research Ethics Committee that approved the study (the West of Scotland Research Ethics Committee), the UK Health Research Authority, and our institutional (University of Leicester) General Research Ethics Committee and Research Governance Office (IRAS project ID: 350113; Research Ethics Committee reference: 25/WS/s0067). This is because the data (both quantitative and qualitative) contain potentially identifying and sensitive participant information, including details of healthcare consultations and health conditions, as well as participant ethnicity, gender, and age, and job role, which in combination can be identifying. We are not permitted by these bodies to share the de-identified data set publicly. Requests for data access may be sent to our departmental email (empathy@leicester.ac.uk) or our institutional ethics email (Ethics@le.ac.uk).   b) Not applicable.   We have updated our Data Availability Statement to match this. |
| **Journal requirements:**  6. We note that there is identifying data in the Supporting Information files <S8 Appendix and S10 Appendix>. Due to the inclusion of these **potentially** identifying data, we have removed this file from your file inventory. Prior to sharing human research participant data, authors should consult with an ethics committee to ensure data are shared in accordance with participant consent and all applicable local laws.    Data sharing should never compromise participant privacy. It is therefore not appropriate to publicly share personally identifiable data on human research participants. The following are examples of data that should not be shared:    -Name, initials, physical address  -Ages more specific than whole numbers  -Internet protocol (IP) address  -Specific dates (birth dates, death dates, examination dates, etc.)  -Contact information such as phone number or email address  -Location data  -ID numbers that seem specific (long numbers, include initials, titled “Hospital ID”) rather than random (small numbers in numerical order)    Data that are not directly identifying may also be inappropriate to share, as in combination they can become identifying. For example, data collected from a small group of participants, vulnerable populations, or private groups should not be shared if they involve indirect identifiers (such as sex, ethnicity, location, etc.) that may risk the identification of study participants.    Additional guidance on preparing raw data for publication can be found in our Data Policy (<https://journals.plos.org/plosone/s/data-availability#loc-human-research-participant-data-and-other-sensitive-data>) and in the following article: <http://www.bmj.com/content/340/bmj.c181.long>.    Please remove or anonymize all personal information (<specific identifying information in file to be removed>), ensure that the data shared are in accordance with participant consent, and re-upload a fully anonymized data set. Please note that spreadsheet columns with personal information must be removed and not hidden as all hidden columns will appear in the published file. | Thank you for raising this important point. In Supporting Information files S8 Appendix and S10 Appendix, we have:   - Replaced ID codes with generic numbers starting from 01 in numerical order. - Replaced specific ages with age brackets. - Removed Geographic Location (in S8 Appendix, this was not applicable to S10 Appendix). - Removed any reference to specific healthcare conditions or professional roles/areas of expertise.   All data is presented in line with guidance from the NHS West of Scotland Research Ethics Committee, the UK Health Research Authority, and University of Leicester General Research Ethics Committee and Research Governance Office, who reviewed and provided ethical approval to conduct and publish this study. We have now re-uploaded the revised Supporting Information file. |
| **Journal requirements:**  7. We note that this data set consists of interview transcripts. Can you please confirm that all participants gave consent for interview transcript to be published?    If they DID provide consent for these transcripts to be published, please also confirm that the transcripts do not contain any potentially identifying information (or let us know if the participants consented to having their personal details published and made publicly available). We consider the following details to be identifying information:  - Names, nicknames, and initials  - Age more specific than round numbers  - GPS coordinates, physical addresses, IP addresses, email addresses  - Information in small sample sizes (e.g. 40 students from X class in X year at X university)  - Specific dates (e.g. visit dates, interview dates)  - ID numbers    Or, if the participants DID NOT provide consent for these transcripts to be published:  - Provide a de-identified version of the data or excerpts of interview responses  - Provide information regarding how these transcripts can be accessed by researchers who meet the criteria for access to confidential data, including:  a) the grounds for restriction  b) the name of the ethics committee, Institutional Review Board, or third-party organization that is imposing sharing restrictions on the data  c) a non-author, institutional point of contact that is able to field data access queries, in the interest of maintaining long-term data accessibility.  d) Any relevant data set names, URLs, DOIs, etc. that an independent researcher would need in order to request your minimal data set.    For further information on sharing data that contains sensitive participant information, please see: <https://journals.plos.org/plosone/s/data-availability#loc-human-research-participant-data-and-other-sensitive-data>    If there are ethical, legal, or third-party restrictions upon your dataset, you must provide all of the following details (<https://journals.plos.org/plosone/s/data-availability#loc-acceptable-data-access-restrictions>):  a) A complete description of the dataset  b) The nature of the restrictions upon the data (ethical, legal, or owned by a third party) and the reasoning behind them  c) The full name of the body imposing the restrictions upon your dataset (ethics committee, institution, data access committee, etc)  d) If the data are owned by a third party, confirmation of whether the authors received any special privileges in accessing the data that other researchers would not have  e) Direct, non-author contact information (preferably email) for the body imposing the restrictions upon the data, to which data access requests can be sent | Thank you.  Participants in this study did not consent to sharing of their interview transcripts, only de-identified excerpts where appropriate. The qualitative data is subject to the same restrictions as described in response to point 5 above.  Our dataset comprises a combination of quantitative and qualitative data from three participant groups: expert panellists (n=9), key stakeholders from each of our respondent groups including patients, practitioners, healthcare educators, carers, and healthcare students (n=35), and patients (n=521). Data from expert panellists includes age, gender, ethnicity, professional role and area of expertise, geographic location, and both Likert-scale scores and free-text comments to assess the relevance of our newly developed Rapid Assessment of Therapeutic Empathy (RATE) scale. Data from key stakeholders from each respondent group includes age, gender, ethnicity, professional role and area of expertise, geographic location, and where relevant health condition, along with interview transcripts describing their thought processes as they complete the newly developed RATE scale, which involves recalling recent healthcare appointments that are likely to be identifying. Data from patients (n=521) includes age, gender, ethnicity, geographic location, details about their last healthcare consultation including the practitioner they saw and where it took place, details about their health condition and/or disability, scores on the RATE scales, the Consultation and Relational Empathy (CARE) measure, and a measure of clinical neutrality. The data are not owned by a third-party organisation.  There are ethical restrictions on sharing our de-identified data set, imposed by the NHS Research Ethics Committee that approved the study (the West of Scotland Research Ethics Committee), the UK Health Research Authority, and our institutional (University of Leicester) General Research Ethics Committee and Research Governance Office (IRAS project ID: 350113; Research Ethics Committee reference: 25/WS/s0067). This is because the data (both quantitative and qualitative) contain potentially identifying and sensitive participant information, including details of healthcare consultations and health conditions, as well as participant ethnicity, gender, and age, and job role, which in combination can be identifying. We are not permitted by these bodies to share the de-identified data set publicly. Requests for data access may be sent to our departmental email (empathy@leicester.ac.uk) or our institutional ethics email (Ethics@le.ac.uk). |
| **Journal requirements:**  8. If the reviewer comments include a recommendation to cite specific previously published works, please review and evaluate these publications to determine whether they are relevant and should be cited. There is no requirement to cite these works unless the editor has indicated otherwise. | Thank you. This is not applicable to our revised manuscript. |
| **Journal requirements:**  9. Please review your reference list to ensure that it is complete and correct. If you have cited papers that have been retracted, please include the rationale for doing so in the manuscript text, or remove these references and replace them with relevant current references. Any changes to the reference list should be mentioned in the rebuttal letter that accompanies your revised manuscript. If you need to cite a retracted article, indicate the article’s retracted status in the References list and also include a citation and full reference for the retraction notice. | Thank you. We have checked our reference list thoroughly. |
| **Editor comment:**  This is an interesting paper on an important topic. The authors have developed a universal single-item clinical empathy scale and conducted psychometric testing of the patient-reported version. It is clear that considerable time and energy have gone into this well-written study, and the reviewers appreciated the quality and relevance of the work. The reviewers have identified some minor revisions that will further strengthen the manuscript. | Thank you for this positive feedback. |
| **Reviewer 1 comments:**  Through this study, the authors have developed a universal single-item clinical empathy scale and validated its patient-reported version. This is a well-written study with a clear and clinically relevant objective that has the potential to facilitate empathy assessment and support improvements in healthcare quality. The authors have successfully captured the key characteristics of empathy within a concise single-item instrument. To further enhance the scale's recognition and accessibility in both clinical practice and future research, the authors may consider adopting a distinctive name or acronym, similar to established instruments such as the CARE Measure or the VAS for pain. For example, "SITE Scale" (Single-Item Therapeutic Empathy Scale) could provide a memorable and easily identifiable naming convention. | Thank you very much for your encouraging comments. We agree that a distinctive acronym would strengthen recognition of our newly developed scale, and have therefore called our scale the “Rapid Assessment of Therapeutic Empathy (RATE) scale”. We have amended the long title to reflect this, as well as:   - The Abstract: lines 24; 46. - The Methods: lines 120-122; 131-134; 148; 151-152; 159; 162; 184; 188; 192; 196; 204; 208; 222; 242; - The Results: lines 257; 259; 270; 279; 287; 289; 298; 304; 322; 325. - The Discussion: lines 328-329; 352; 381; 384; 388; 399. - Figure 2 title: line 264. - Figure 3 title: line 296. - Supporting information title, sub-headings. |
| **Reviewer 1 comments:**  This study has also validated the patient-reported version of the scale, and I look forward to future validation of the practitioner-, observer-, and student-reported versions. I would also like to point out that the visual presentation of the correlation data could be improved. Figure 4A and 4B use different scales despite having identical dimensions, which may inadvertently encourage direct visual comparison. Possible alternatives include using different widths for the two figures or placing Figure 4C, which is visually distinct, between Figures 4A and 4B to provide clearer visual separation. | Thank you. We have altered the width of Figure 4b to avoid direct visual comparison, as you helpfully suggested. |
| **Reviewer 2 comments:**  Thank you for this insightful and well written paper. I outline a few suggestions for improvement below.  Introduction  Consider expanding the second paragraph to discuss the concept of therapeutic empathy in more fully. Perhaps mention the cognitive, emotional and relational aspects of therapeutic empathy and expand on the six components. | Thank you for your positive feedback.  We have expanded the second paragraph of our Introduction (lines 58-64) to expand the description of each component of therapeutic empathy and note that they entail both cognitive and affective dimensions. |
| **Reviewer 2 comments:**  Methods  The methods are well described seem appropriate to address the research questions.  The sampling strategy is well suited to the study objectives and well described. I particularly liked the efforts taken to prioritise inclusion of neglected and underserved groups in the patient testing.  The authors mention use of plain English, pictorial, text based and unipolar versions of the scale. Were there any modifications to include people who did not speak English as a first language, non-literate people or those living with learning disability? If so it would be good to include more details of this. | Thank you.  Thank you for acknowledging our efforts here.  In the methods, we have clarified that this study was focused on the development and validation of an English-language version of the scale, in line with best-practice guidance, on lines 182-183. In the Discussion, we have acknowledged that the scale was administered to participants in English, without recording participants’ native language, as a limitation (lines 377-379). We have also noted that validation in patients with learning disabilities, and of the scale in different languages, would be beneficial for future research (line 390). |
| **Reviewer 2 comments:**  Results  The findings are discussed well and in detail. However, it would more readable to have the respondent characteristics and statistical test values for convergent validity presented as tables rather than as blocks of text. | Thank you. We have converted large chunks of text describing participants’ characteristics and the results of our tests of convergent validity by subgroup into tables, please see lines 298-308. |
| **Reviewer 2 comments:**  Discussion  The discussion section is insightful, and the authors have contextualised the findings well within the broader literature. The implications for research and practice are discussed appropriately. There is clearly a lot of use for a validated scale such as this across clinical education, research and quality improvement. | Thank you very much for your kind words, and for recognising the potential of our scale. |
