# Supplementary material for: Rapid Assessment of Therapeutic Empathy (RATE): Development of a universal single-item empathy scale and validation of the patient-reported version
Source: PLoS One. 2026 Sep 9;21(9):e0346994. doi: 10.1371/journal.pone.0346994 (PMC13557358; doi:10.1371/journal.pone.0346994)
Supplement: Peer review history

# Peer review: Decision Letter 0

**DOI:** 10.1371/journal.pone.0346994.r001

**Authors:** Ipek Gonullu

20 Jul 2026

Dear Dr. Bennett-Weston,

Thank you for submitting your manuscript to PLOS ONE. After careful consideration, we feel that it has merit but does not fully meet PLOS ONE’s publication criteria as it currently stands. Therefore, we invite you to submit a revised version of the manuscript that addresses the points raised during the review process.

==============================

This is an interesting paper on an important topic. The authors have developed a universal single-item clinical empathy scale and conducted psychometric testing of the patient-reported version. It is clear that considerable time and energy have gone into this well-written study, and the reviewers appreciated the quality and relevance of the work. The reviewers have identified some minor revisions that will further strengthen the manuscript.

==============================

Please submit your revised manuscript by Sep 03 2026 11:59PM. If you will need more time than this to complete your revisions, please reply to this message or contact the journal office at plosone@plos.org. When you're ready to submit your revision, log on to https://www.editorialmanager.com/pone/ and select the 'Submissions Needing Revision' folder to locate your manuscript file.

- A letter that responds to each point raised by the academic editor and reviewer(s). You should upload this letter as a separate file labeled 'Response to Reviewers'.
- A marked-up copy of your manuscript that highlights changes made to the original version. You should upload this as a separate file labeled 'Revised Manuscript with Track Changes'.
- An unmarked version of your revised paper without tracked changes. You should upload this as a separate file labeled 'Manuscript'.

If you would like to make changes to your financial disclosure, please include your updated statement in your cover letter. Guidelines for resubmitting your figure files are available below the reviewer comments at the end of this letter.

If applicable, we recommend that you deposit your laboratory protocols in protocols.io to enhance the reproducibility of your results. Protocols.io assigns your protocol its own identifier (DOI) so that it can be cited independently in the future. For instructions see: https://journals.plos.org/plosone/s/submission-guidelines#loc-laboratory-protocols. Additionally, PLOS ONE offers an option for publishing peer-reviewed Lab Protocol articles, which describe protocols hosted on protocols.io. Read more information on sharing protocols at https://plos.org/protocols?utm_medium=editorial-email&utm_source=authorletters&utm_campaign=protocols.

As the corresponding author, your ORCID iD is verified in the submission system and will appear in the published article. PLOS supports the use of ORCID, and we encourage all coauthors to register for an ORCID iD and use it as well. Please encourage your coauthors to verify their ORCID iD within the submission system before final acceptance, as unverified ORCID iDs will not appear in the published article. *Only*  the individual author can complete the verification step; PLOS staff *cannot*  verify ORCID iDs on behalf of authors.

We look forward to receiving your revised manuscript.

Kind regards,

Ipek Gonullu, M.D., Ph.D.

Academic Editor

PLOS One

**Journal Requirements:**

1. When submitting your revision, we need you to address these additional requirements.

Please ensure that your manuscript meets PLOS ONE's style requirements, including those for file naming. The PLOS ONE style templates can be found at

https://journals.plos.org/plosone/s/file?id=wjVg/PLOSOne_formatting_sample_main_body.pdf and

https://journals.plos.org/plosone/s/file?id=ba62/PLOSOne_formatting_sample_title_authors_affiliations.pdf

2. We note that the grant information you provided in the ‘Funding Information’ and ‘Financial Disclosure’ sections do not match.

When you resubmit, please ensure that you provide the correct grant numbers for the awards you received for your study in the ‘Funding Information’ section.

3. Thank you for stating in your Funding Statement:

JH, ABW, BIW, and PO are funded by the Stoneygate Trust (no grant reference). KK is supported by the National Institute for Health Research (NIHR; no grant reference). DN was funded by NIHR (grant reference: NIHR305951). The funders had no role in developing the protocol.

Please provide an amended statement that declares *all* the funding or sources of support (whether external or internal to your organization) received during this study, as detailed online in our guide for authors at http://journals.plos.org/plosone/s/submit-now.  Please also include the statement “There was no additional external funding received for this study.” in your updated Funding Statement.

Please include your amended Funding Statement within your cover letter. We will change the online submission form on your behalf.

4. Please expand the acronym “JH, ABW, BIW, PO KK and DN” (as indicated in your financial disclosure) so that it states the name of your funders in full.

This information should be included in your cover letter; we will change the online submission form on your behalf.

5. We note that you have indicated that there are restrictions to data sharing for this study. For studies involving human research participant data or other sensitive data, we encourage authors to share de-identified or anonymized data. However, when data cannot be publicly shared for ethical reasons, we allow authors to make their data sets available upon request. For information on unacceptable data access restrictions, please see http://journals.plos.org/plosone/s/data-availability#loc-unacceptable-data-access-restrictions.

Before we proceed with your manuscript, please address the following prompts:

a) If there are ethical or legal restrictions on sharing a de-identified data set, please explain them in detail (e.g., data contain potentially identifying or sensitive patient information, data are owned by a third-party organization, etc.) and who has imposed them (e.g., a Research Ethics Committee or Institutional Review Board, etc.). Please also provide contact information for a data access committee, ethics committee, or other institutional body to which data requests may be sent.

b) If there are no restrictions, please upload the minimal anonymized data set necessary to replicate your study findings to a stable, public repository and provide us with the relevant URLs, DOIs, or accession numbers. Please see http://www.bmj.com/content/340/bmj.c181.long for guidelines on how to de-identify and prepare clinical data for publication. For a list of recommended repositories, please see https://journals.plos.org/plosone/s/recommended-repositories. You also have the option of uploading the data as Supporting Information files, but we would recommend depositing data directly to a data repository if possible.

Please update your Data Availability statement in the submission form accordingly.

6. We note that there is identifying data in the Supporting Information files <S8 Appendix and S10 Appendix>. Due to the inclusion of these potentially identifying data, we have removed this file from your file inventory. Prior to sharing human research participant data, authors should consult with an ethics committee to ensure data are shared in accordance with participant consent and all applicable local laws.

Data sharing should never compromise participant privacy. It is therefore not appropriate to publicly share personally identifiable data on human research participants. The following are examples of data that should not be shared:

-Name, initials, physical address

-Ages more specific than whole numbers

-Internet protocol (IP) address

-Specific dates (birth dates, death dates, examination dates, etc.)

-Contact information such as phone number or email address

-Location data

-ID numbers that seem specific (long numbers, include initials, titled “Hospital ID”) rather than random (small numbers in numerical order)

Data that are not directly identifying may also be inappropriate to share, as in combination they can become identifying. For example, data collected from a small group of participants, vulnerable populations, or private groups should not be shared if they involve indirect identifiers (such as sex, ethnicity, location, etc.) that may risk the identification of study participants.

Additional guidance on preparing raw data for publication can be found in our Data Policy (https://journals.plos.org/plosone/s/data-availability#loc-human-research-participant-data-and-other-sensitive-data) and in the following article: http://www.bmj.com/content/340/bmj.c181.long.

Please remove or anonymize all personal information (<specific identifying information in file to be removed>), ensure that the data shared are in accordance with participant consent, and re-upload a fully anonymized data set. Please note that spreadsheet columns with personal information must be removed and not hidden as all hidden columns will appear in the published file.

7. We note that this data set consists of interview transcripts. Can you please confirm that all participants gave consent for interview transcript to be published?

If they DID provide consent for these transcripts to be published, please also confirm that the transcripts do not contain any potentially identifying information (or let us know if the participants consented to having their personal details published and made publicly available). We consider the following details to be identifying information:

- Names, nicknames, and initials

- Age more specific than round numbers

- GPS coordinates, physical addresses, IP addresses, email addresses

- Information in small sample sizes (e.g. 40 students from X class in X year at X university)

- Specific dates (e.g. visit dates, interview dates)

- ID numbers

Or, if the participants DID NOT provide consent for these transcripts to be published:

- Provide a de-identified version of the data or excerpts of interview responses

- Provide information regarding how these transcripts can be accessed by researchers who meet the criteria for access to confidential data, including:

a) the grounds for restriction

b) the name of the ethics committee, Institutional Review Board, or third-party organization that is imposing sharing restrictions on the data

c) a non-author, institutional point of contact that is able to field data access queries, in the interest of maintaining long-term data accessibility.

d) Any relevant data set names, URLs, DOIs, etc. that an independent researcher would need in order to request your minimal data set.

For further information on sharing data that contains sensitive participant information, please see: https://journals.plos.org/plosone/s/data-availability#loc-human-research-participant-data-and-other-sensitive-data

If there are ethical, legal, or third-party restrictions upon your dataset, you must provide all of the following details (https://journals.plos.org/plosone/s/data-availability#loc-acceptable-data-access-restrictions):

a) A complete description of the dataset

b) The nature of the restrictions upon the data (ethical, legal, or owned by a third party) and the reasoning behind them

c) The full name of the body imposing the restrictions upon your dataset (ethics committee, institution, data access committee, etc)

d) If the data are owned by a third party, confirmation of whether the authors received any special privileges in accessing the data that other researchers would not have

e) Direct, non-author contact information (preferably email) for the body imposing the restrictions upon the data, to which data access requests can be sent

8. If the reviewer comments include a recommendation to cite specific previously published works, please review and evaluate these publications to determine whether they are relevant and should be cited. There is no requirement to cite these works unless the editor has indicated otherwise. 

9. Please review your reference list to ensure that it is complete and correct. If you have cited papers that have been retracted, please include the rationale for doing so in the manuscript text, or remove these references and replace them with relevant current references. Any changes to the reference list should be mentioned in the rebuttal letter that accompanies your revised manuscript. If you need to cite a retracted article, indicate the article’s retracted status in the References list and also include a citation and full reference for the retraction notice.

**Additional Editor Comments:**

This is an interesting paper on an important topic. The authors have developed a universal single-item clinical empathy scale and conducted psychometric testing of the patient-reported version. It is clear that considerable time and energy have gone into this well-written study, and the reviewers appreciated the quality and relevance of the work. The reviewers have identified some minor revisions that will further strengthen the manuscript.

Reviewers' comments:

**Comments to the Author**

1. Is the manuscript technically sound, and do the data support the conclusions?

Reviewer #1: Yes

Reviewer #2: Yes

**********

2. Has the statistical analysis been performed appropriately and rigorously?

Reviewer #1: Yes

Reviewer #2: I Don't Know

**********

3. Have the authors made all data underlying the findings in their manuscript fully available?

The PLOS Data policy

Reviewer #1: Yes

Reviewer #2: No

**********

4. Is the manuscript presented in an intelligible fashion and written in standard English?

Reviewer #1: Yes

Reviewer #2: Yes

**********

**Reviewer #1:**  Through this study, the authors have developed a universal single-item clinical empathy scale and validated its patient-reported version. This is a well-written study with a clear and clinically relevant objective that has the potential to facilitate empathy assessment and support improvements in healthcare quality. The authors have successfully captured the key characteristics of empathy within a concise single-item instrument. To further enhance the scale's recognition and accessibility in both clinical practice and future research, the authors may consider adopting a distinctive name or acronym, similar to established instruments such as the CARE Measure or the VAS for pain. For example, "SITE Scale" (Single-Item Therapeutic Empathy Scale) could provide a memorable and easily identifiable naming convention

This study has also validated the patient-reported version of the scale, and I look forward to future validation of the practitioner-, observer-, and student-reported versions. I would also like to point out that the visual presentation of the correlation data could be improved. Figure 4A and 4B use different scales despite having identical dimensions, which may inadvertently encourage direct visual comparison. Possible alternatives include using different widths for the two figures or placing Figure 4C, which is visually distinct, between Figures 4A and 4B to provide clearer visual separation.

**Reviewer #2:**  Thank you for this insightful and well written paper. I outline a few suggestions for improvement below.

Introduction

Consider expanding the second paragraph to discuss the concept of therapeutic empathy in more fully. Perhaps mention the cognitive, emotional and relational aspects of therapeutic empathy and expand on the six components.

Methods

The methods are well described seem appropriate to address the research questions.

The sampling strategy is well suited to the study objectives and well described. I particularly liked the efforts taken to prioritise inclusion of neglected and underserved groups in the patient testing.

The authors mention use of plain English, pictorial, text based and unipolar versions of the scale. Were there any modifications to include people who did not speak English as a first language, non-literate people or those living with learning disability? If so it would be good to include more details of this.

Results

The findings are discussed well and in detail. However, it would more readable to have the respondent characteristics and statistical test values for convergent validity presented as tables rather than as blocks of text.

Discussion

The discussion section is insightful, and the authors have contextualised the findings well within the broader literature. The implications for research and practice are discussed appropriately. There is clearly a lot of use for a validated scale such as this across clinical education, research and quality improvement.

**********

what does this mean?). If published, this will include your full peer review and any attached files.

If you choose “no”, your identity will remain anonymous but your review may still be made public.

**Do you want your identity to be public for this peer review?**  For information about this choice, including consent withdrawal, please see our Privacy Policy

Reviewer #1: No

Reviewer #2: **Yes:** Advaith Gummaraju

**********

[NOTE: If reviewer comments were submitted as an attachment file, they will be attached to this email and accessible via the submission site. Please log into your account, locate the manuscript record, and check for the action link "View Attachments". If this link does not appear, there are no attachment files.]

To ensure your figures meet our technical requirements, please review our figure guidelines: https://journals.plos.org/plosone/s/figures

You may also use PLOS’s free figure tool, NAAS, to help you prepare publication quality figures: https://journals.plos.org/plosone/s/figures#loc-tools-for-figure-preparation.

NAAS will assess whether your figures meet our technical requirements by comparing each figure against our figure specifications.

# Author comment: Author response to Decision Letter 1

**DOI:** 10.1371/journal.pone.0346994.r002

22 Jul 2026

We would like to thank the reviewers for their thoughtful and scholarly comments on our manuscript. Please see the uploaded "Response to Reviewers" document, and the responses below for a description of how we have addressed these comments:

Reviewer 1: "Through this study, the authors have developed a universal single-item clinical empathy scale and validated its patient-reported version. This is a well-written study with a clear and clinically relevant objective that has the potential to facilitate empathy assessment and support improvements in healthcare quality. The authors have successfully captured the key characteristics of empathy within a concise single-item instrument. To further enhance the scale's recognition and accessibility in both clinical practice and future research, the authors may consider adopting a distinctive name or acronym, similar to established instruments such as the CARE Measure or the VAS for pain. For example, "SITE Scale" (Single-Item Therapeutic Empathy Scale) could provide a memorable and easily identifiable naming convention."

Author response: "Thank you very much for your encouraging comments. We agree that a distinctive acronym would strengthen recognition of our newly developed scale, and have therefore called our scale the “Rapid Assessment of Therapeutic Empathy (RATE) scale”. We have amended the long title to reflect this, as well as:

• The Abstract: lines 24; 46.

• The Methods: lines 120-122; 131-134; 148; 151-152; 159; 162; 184; 188; 192; 196; 204; 208; 222; 242;

• The Results: lines 257; 259; 270; 279; 287; 289; 298; 304; 322; 325.

• The Discussion: lines 328-329; 352; 381; 384; 388; 399.

• Figure 2 title: line 264.

• Figure 3 title: line 296.

• Supporting information title, sub-headings."

Reviewer 1: "This study has also validated the patient-reported version of the scale, and I look forward to future validation of the practitioner-, observer-, and student-reported versions. I would also like to point out that the visual presentation of the correlation data could be improved. Figure 4A and 4B use different scales despite having identical dimensions, which may inadvertently encourage direct visual comparison. Possible alternatives include using different widths for the two figures or placing Figure 4C, which is visually distinct, between Figures 4A and 4B to provide clearer visual separation."

Author response: "Thank you. We have altered the width of Figure 4b to avoid direct visual comparison, as you helpfully suggested."

Reviewer 2: "Thank you for this insightful and well written paper. I outline a few suggestions for improvement below.

Introduction

Consider expanding the second paragraph to discuss the concept of therapeutic empathy in more fully. Perhaps mention the cognitive, emotional and relational aspects of therapeutic empathy and expand on the six components."

Author response: "Thank you for your positive feedback.

We have expanded the second paragraph of our Introduction (lines 58-64) to expand the description of each component of therapeutic empathy and note that they entail both cognitive and affective dimensions. "

Reviewer 2: "Methods

The methods are well described seem appropriate to address the research questions.

The sampling strategy is well suited to the study objectives and well described. I particularly liked the efforts taken to prioritise inclusion of neglected and underserved groups in the patient testing.

The authors mention use of plain English, pictorial, text based and unipolar versions of the scale. Were there any modifications to include people who did not speak English as a first language, non-literate people or those living with learning disability? If so it would be good to include more details of this."

Author response: "Thank you.

Thank you for acknowledging our efforts here.

In the methods, we have clarified that this study was focused on the development and validation of an English-language version of the scale, in line with best-practice guidance, on lines 182-183. In the Discussion, we have acknowledged that the scale was administered to participants in English, without recording participants’ native language, as a limitation (lines 377-379). We have also noted that validation in patients with learning disabilities, and of the scale in different languages, would be beneficial for future research (line 390)."

Reviewer 2: "Results

The findings are discussed well and in detail. However, it would more readable to have the respondent characteristics and statistical test values for convergent validity presented as tables rather than as blocks of text."

Author Response: "Thank you. We have converted large chunks of text describing participants’ characteristics and the results of our tests of convergent validity by subgroup into tables, please see lines 298-308."

Reviewer 2: "Discussion

The discussion section is insightful, and the authors have contextualised the findings well within the broader literature. The implications for research and practice are discussed appropriately. There is clearly a lot of use for a validated scale such as this across clinical education, research and quality improvement."

Author response: "Thank you very much for your kind words, and for recognising the potential of our scale."

Attachment Submitted filename: Response_to_Reviewers.docx

# Editor report: Decision Letter 1

**DOI:** 10.1371/journal.pone.0346994.r003

**Authors:** Ipek Gonullu

26 Jul 2026

Rapid Assessment of Therapeutic Empathy (RATE): Development of a universal single-item empathy scale and validation of the patient-reported version

PONE-D-26-14659R1

Dear Dr. Bennett-Weston,

We’re pleased to inform you that your manuscript has been judged scientifically suitable for publication and will be formally accepted for publication once it meets all outstanding technical requirements.

Within one week, you’ll receive an e-mail detailing the required amendments. When these have been addressed, you’ll receive a formal acceptance letter and your manuscript will be scheduled for publication.

An invoice will be generated when your article is formally accepted. Please note, if your institution has a publishing partnership with PLOS and your article meets the relevant criteria, all or part of your publication costs will be covered. Please make sure your user information is up-to-date by logging into Editorial Manager at Editorial Manager® and clicking the ‘Update My Information' link at the top of the page. For questions related to billing, please contact billing support.

If your institution or institutions have a press office, please notify them about your upcoming paper to help maximize its impact. If they’ll be preparing press materials, please inform our press team as soon as possible -- no later than 48 hours after receiving the formal acceptance. Your manuscript will remain under strict press embargo until 2 pm Eastern Time on the date of publication. For more information, please contact onepress@plos.org.

Kind regards,

Ipek Gonullu, M.D., Ph.D.

Academic Editor

PLOS One

Additional Editor Comments (optional):

I thank the authors for addressing the reviewers’ comments. In this version of the manuscript, missing points have been successfully completed and the manuscript is revised according to the comments.

Reviewers' comments:

# Editor report: Acceptance letter

**DOI:** 10.1371/journal.pone.0346994.r004

**Authors:** Ipek Gonullu

PONE-D-26-14659R1

PLOS One

Dear Dr. Bennett-Weston,

I'm pleased to inform you that your manuscript has been deemed suitable for publication in PLOS One. Congratulations! Your manuscript is now being handed over to our production team.

At this stage, our production department will prepare your paper for publication. This includes ensuring the following:

* All references, tables, and figures are properly cited

* All relevant supporting information is included in the manuscript submission,

* There are no issues that prevent the paper from being properly typeset

You will receive further instructions from the production team, including instructions on how to review your proof when it is ready. Please keep in mind that we are working through a large volume of accepted articles, so please give us a few days to review your paper and let you know the next and final steps.

Lastly, if your institution or institutions have a press office, please let them know about your upcoming paper now to help maximize its impact. If they'll be preparing press materials, please inform our press team within the next 48 hours. Your manuscript will remain under strict press embargo until 2 pm Eastern Time on the date of publication. For more information, please contact onepress@plos.org.

You will receive an invoice from PLOS for your publication fee after your manuscript has reached the completed accept phase. If you receive an email requesting payment before acceptance or for any other service, this may be a phishing scheme. Learn how to identify phishing emails and protect your accounts at https://explore.plos.org/phishing.

If we can help with anything else, please email us at customercare@plos.org.

Thank you for submitting your work to PLOS One and supporting open access.

Kind regards,

PLOS ONE Editorial Office Staff

on behalf of

Associate Professor Ipek Gonullu

Academic Editor

PLOS One
